# The fecal microbiota of patients with pancreatic ductal adenocarcinoma and autoimmune pancreatitis characterized by metagenomic sequencing

**DOI:** 10.1186/s12967-021-02882-7

**Published:** 2021-05-18

**Authors:** Wenli Zhou, De Zhang, Zhengpeng Li, Huiqing Jiang, Jingnan Li, Rongrong Ren, Xuefeng Gao, Jianfeng Li, Xin Wang, Weifeng Wang, Yunsheng Yang

**Affiliations:** 1 School of Medicine, Nankai University, Tianjin, 300190 China; 2 Micriobiota Division, Department of Gastroenterology and Hepatology, The First Medical Center, Chinese PLA General Hospital, Beijing, 100853 China; 3 School of Medicine, Chinese PLA General Hospital, Beijing, 100853 China; 4 Institute of Plant Protection and Microbiology, Zhejiang Academy of Agricultural Sciences, Hangzhou, 310021 China; 5 Department of Gastroenterology, The Second Affiliated Hospital of Hebei Medical University, Shijiazhuang, 050000 China; 6 Department of Gastroenterology, Peking Union Hospital, Beijing, 100005 China; 7 Department of Gastroenterology and Hepatology, Shenzhen University General Hospital, Shenzhen, 518055 China; 8 Clinical Medical Academy, Shenzhen University, Shenzhen, 518060 China; 9 National Clinical Research Center for Geriatric Diseases, Chinese PLA General Hospital, Beijing, 100853 China

**Keywords:** Pancreatic ductal adenocarcinoma, Autoimmune pancreatitis, Fecal microbiota, Metagenomic sequencing, Butyrate

## Abstract

**Background:**

The fecal microbiota in pancreatic ductal adenocarcinoma (PDAC) and in autoimmune pancreatitis (AIP) patients remains largely unknown. We aimed to characterize the fecal microbiota in patients with PDAC and AIP, and explore the possibility of fecal microbial biomarkers for distinguishing PDAC and AIP.

**Methods:**

32 patients with PDAC, 32 patients with AIP and 32 age- and sex-matched healthy controls (HC) were recruited and the fecal microbiotas were analyzed through high-throughput metagenomic sequencing. Alterations of fecal short-chain fatty acids were measured using gas chromatographic method.

**Results:**

Principal coordinate analysis (PCoA) revealed that microbial compositions differed significantly between PDAC and HC samples; whereas, AIP and HC individuals tended to cluster together. Significant reduction of phylum Firmicutes (especially butyrate-producing bacteria, including *Eubacterium rectale*, *Faecalibacterium prausnitzii* and *Roseburia intestinalis*) and significant increase of phylum Proteobacteria (especially Gammaproteobacteria) were observed only among PDAC samples. At species level, when compared with HC samples, we revealed 24 and 12 differently enriched bacteria in PDAC and AIP, respectively. Functional analysis showed a depletion of short-chain fatty acids synthesis associated KO modules (e.g. Wood-Ljungdahl pathway) and an increase of KO modules associated with bacterial virulence (e.g. type II general secretion pathway). Consistent with the downregulation of butyrate-producing bacteria, gas chromatographic analysis showed fecal butyrate content was significantly decreased in PDAC group*. Eubacterium rectale*, *Eubacterium ventrisum* and *Odoribacter splanchnicus* were among the most important biomarkers in distinguishing PDAC from HC and from AIP individuals. Receiver Operating Characteristic analysis showed areas under the curve of 90.74% (95% confidence interval [CI] 86.47–100%), 88.89% (95% CI 73.49–100%), and 76.54% (95% CI 52.5–100%) for PDAC/HC, PDAC/AIP and AIP/HC, respectively.

**Conclusions:**

In conclusion, alterations in fecal microbiota and butyrate of patients with PDAC suggest an underlying role of gut microbiota for the pathogenesis of PDAC. Fecal microbial and butyrate as potential biomarkers may facilitate to distinguish patients with PDAC from patients with AIP and HCs which worth further validation.

**Supplementary Information:**

The online version contains supplementary material available at 10.1186/s12967-021-02882-7.

## Introduction

Pancreatic cancer is one of the most common cause of cancer death and leads to an estimated 227,000 deaths annually worldwide [1], and more than 80% of the pancreatic malignancy are the pancreatic ductal adenocarcinomas (PDAC). Because of the lack of an effective early detection methods, 80–85% of patients are past the optimal window for surgery once diagnosed, together with PDAC’s highly invasive behavior and poor sensitivity to conventional and targeted therapies, leading to a very low 5-year survival rate of only 5% among patients diagnosed with PDAC [2]. Therefore, the pathogenesis, the new diagnostic strategies and preventive therapeutic means must be explored for PDAC.

Autoimmune pancreatitis (AIP), which belongs to the spectrum of immunoglobulin G4 (IgG4)-related diseases, is a chronic inflammatory disease of the pancreas, likely with an autoimmune etiology [3]. To date, the AIP and IgG4-related disease pathogeneses are largely unknown. Evidence suggests that *Helicobacter pylori* plays a role in the AIP pathogenesis via molecular mimicry [4]. AIP is mostly accompanied by an expanded pancreas; however, AIP remains challenging to diagnose at an early stage or to distinguish between PDAC and AIP patients via imaging, which can result in unnecessary surgical resection when PDAC is suspected [5]. Thus, new effective, noninvasive approaches for differentiating AIP from PDAC are urgently needed.

The human gut is a large reservoir of microbes. The Bacteroidetes and Firmicutes phyla are the most dominant, followed by Actinobacteria, Proteobacteria and Verrucomicrobia [6]. Evidence suggests that the gut microbiota and inflammation play roles in many diseases, including several cancers such as colorectal cancer [7]. Patients with chronic pancreatitis present a high risk of PDAC, suggesting that inflammation may play a role in PDAC. The relationship between the oral microbiota and PDAC has been reported in several studies [2, 8, 9]; however, little is known about the composition and role of the gut microbiota in PDAC and in AIP. Here, we thoroughly evaluated for the first time the compositional and butyric alterations in the fecal microbiota in PDAC and AIP patients, and investigated the possibility of gut microbial biomarkers as noninvasive methods for diagnosing PDAC and differentiating between PDAC and AIP.

## Materials and methods

### Patient cohorts

Ninety-six participants were enrolled in our study, including 32 patients newly diagnosed with PDCA from Chinese PLA General Hospital, 32 patients with AIP and 32 age-and sex-matched healthy controls (HCs). All 32 patients with PDCA were diagnosed via surgery and pathology. AIP was diagnosed according to the international consensus diagnostic criteria for AIP proposed by the International Association of Pancreatology and only patients diagnosed as type 1 AIP characterized by elevated serum IgG4 levels were enrolled in our study [10]. The subjects in HC group were selected from individuals who visited the Chinese PLA General Hospital for their health check and pass the exclusion criteria described below. Exclusion criteria for all participants included irritable bowel disease, celiac disease, other cancers, and autoimmune diseases (except AIP). Participants had not been administered antibiotics, antifungals, probiotics or prebiotics for at least 2 months before sampling. Table 1 shows the details of all study participants. Fresh fecal samples were collected and transported to our laboratory in an ice bag within 2 h and then stored at −> 80 °C until testing.

**Table 1:** Demographic and clinical details of samples

Factor	PDAC (n = 32)	HC (n = 32)	AIP (n = 32)
Age	59.31 ± 9.53	58.63 ± 10.28	58.83 ± 9.76
Gender (F/n)	21.87% (7)	18.75% (6)	18.75% (6)
BMI	23.21 ± 3.24	23.18 ± 3.07	24.37 ± 3.09
Hypertension (n)	34.38% (11)	25% (8)	6.25% (2)
Diabetes (n)	31.25% (10)	16% (5)	28.13% (9)
Jaundice (n)	25.00% (8)	0 (0)	0 (0)
Tumor location (n)			
Pancreatic head	40.63% (13)	–	–
Pancreatic neck	12.50% (4)	–	–
Pancreatic body	3.13% (1)	–	–
Pancreatic tail	6.25% (2)	–	–
Pancreatic head and body	12.50% (4)	–	–
Pancreatic body and tail	25.00% (8)	–	–
TNS staging (n)			
I	37.50% (12)	–	–
II	28.13% (9)	–	–
III	18.75% (6)	–	–
IV	15.63% (5)	–	–
CA-19–9 u/ml	137.10(15.54–379.60)	–	–

Data are expressed as mean ± SD or median (1st-3st quartile) according to the normality of distribution. F: female, PDAC: pancreatic ductal adenocarcinoma, AIP: autoimmune pancreatitis, HC: healthy control

### Metagenomics sequencing

Fecal samples were processed for DNA extraction (QIAampPowerFecal Pro DNA Kit), quality control and DNA library construction (concentration > 3 nM). The library quality was controlled by Qubit2.0 (Thermo Fisher Scientific), qSep100 (BiOptic) and q-PCR (Thermo Fisher Scientific). Metagenomic shotgun sequencing was performed using an Illumina HiSeq Platform. Original sequencing reads (607.6G) were obtained with an average of 45,304,701 reads per sample. The numbers of reads per sample ranged from 30,751,784 to 73,847,648. High-quality clean reads were obtained by removing reads of less than 30 bp, low-quality reads (< 20), and contaminated human reads (Additional file 1). Using SOAPdenovo (v2.04, parameters “all -D1 -M3 -L500”), the high-quality clean reads were then de novo assembled into contigs (Additional file 2). Genes of the assembled contigs with more than 500 bp were predicted using MetaGeneMark [11]. The predicted genes were clustered to create a non-redundant gene catalogue using CD-HIT [12], with cutoffs of 90% overlap and 95% identity. Relative gene abundances were determined by aligning high-quality clean reads to the non-redundant gene catalogue using BWA alignment tool, following normalization using the reads per kilobase per million (RPKM) method.

### Taxonomic annotation and functional analysis of metagenomic sequences

MetaPhlAn2.0 [13], a method using clade-specific marker genes, was used to perform metagenomic taxonomic profiling. Relative abundances at every taxonomic level were estimated based on reads counts. Moreover, metagenomic species (MGS) profiling was performed to complement MetaPhlAn2.0, following the method described by Nielsen et al. [14] without using reference genomes. Here, co-abundance gene groups (CAGs) with more than 500 genes (also called MGS) were used for further annotation. MGSs were annotated to a bacterial species with a threshold of > 50% of the genes in any MGSs assigned to the integrated gut genome dataset constructed by Nanfach et al. [15]. Co-occurrence analysis was performed using the CoNet app as previously described [16] and visualized usingCytoscape5. × (app version).

Genes in the non-redundant gene catalogue were translated into amino acids for further alignment against the proteins/domains in the Kyoto Encyclopedia of Genes and Genomes (KEGG) ortholog (KO) database using DIAMOND (v0.7.9.58; parameters: blast -v -sensitive -k 10). Each gene was then assigned to a KO group filtered by e-value < 1e^−5^ and percent identity > 70%. Reporter scores were calculated for each KO module per the method in [17]. The differentially enriched KO modules were identified with a threshold of ≥ 2.3 or ≤ − 2.3.

We compared the three groups’ potentials to produce butyrate per the method of Vital et al. [18]. Briefly, high-quality clean reads were aligned against the database established by Vital et al. [18] with ≥ 70 bp alignment and ≥ 80% identity. The abundances of the butyrate-producing pathways (the acetyl-CoA, lysine, 4-aminobutyrate, and glutarate pathways) and the involved genes were then calculated following the steps described by Vital et al. [18]. We evaluated the three groups’ potentials to produce polyamines with HUMAnN2 which classifies the reads into MetaCyc pathways [19].

### Determination of short-chain fatty acids (SCFA)

The concentrations of SCFA in fecal samples was evaluated by gas chromatography.

### Classifier

The random forest method was performed, and the dataset was partitioned into the training set (70%) and testing set (30%). XGBOOST was applied to select features and the leave-one-out-cross-validation method was performed to select the prediction model. The performance was evaluated using the receiver operation characteristic curve (ROC) and area under the ROC curve (AUC). Thresholds of each bacteria by which ROC analysis was performed was set at 0.5.

### Linear discriminant analysis effect size (LEfSe) analysis

Characterized by combination of non-parametric test and biological significance, LEfSe is a robust tool for identifying biomarkers from microbial metagenome data [20]. Thus, LEfSe analysis was performed as an evaluation of robustness of classifier constructed through random forest method. Here, bacterial species with LDA score > 2 and P < 0.05 were considered to be significant.

### Statistical analysis

Continuous variables were expressed as mean ± SD for normal distribution and median with interquartile range for non-normal distribution. Discrete variables were expressed as percentages. Normal distribution was tested by one-way ANOVA test. Non-normal distribution was tested by the Kruskal–Wallis test followed by Steel–Dwass test for pairwise comparison. Statistical analyses were performed using R software packages or SPSS19.0 (IBM). P-values were corrected by Benjamini–Hochberg method for multiple comparisons.

The Bray–Curtis distance-based principal coordinate analysis (PCoA) was performed at the species level to assess species composition dissimilarity. Permutational multivariate analysis of variance (PERMANOVA) [21] (‘*Adonis*’ function, vegan package, R; 1000 permutations) was used to assess the influence of phenotypic variances to sample differences.

## Results

### Analysis of the microbial community structure in the PDAC and AIP groups

We constructed a non-redundant gene catalogue from 96 participants, which contained 2585854, 2522210 and 2538553 genes from the PDAC, AIP and HC groups, respectively. The three groups shared 1899961 genes, with 240991, 194826 and 219119 specific genes in the PDAC, AIP and HC groups, respectively.

**Fig. 1:**
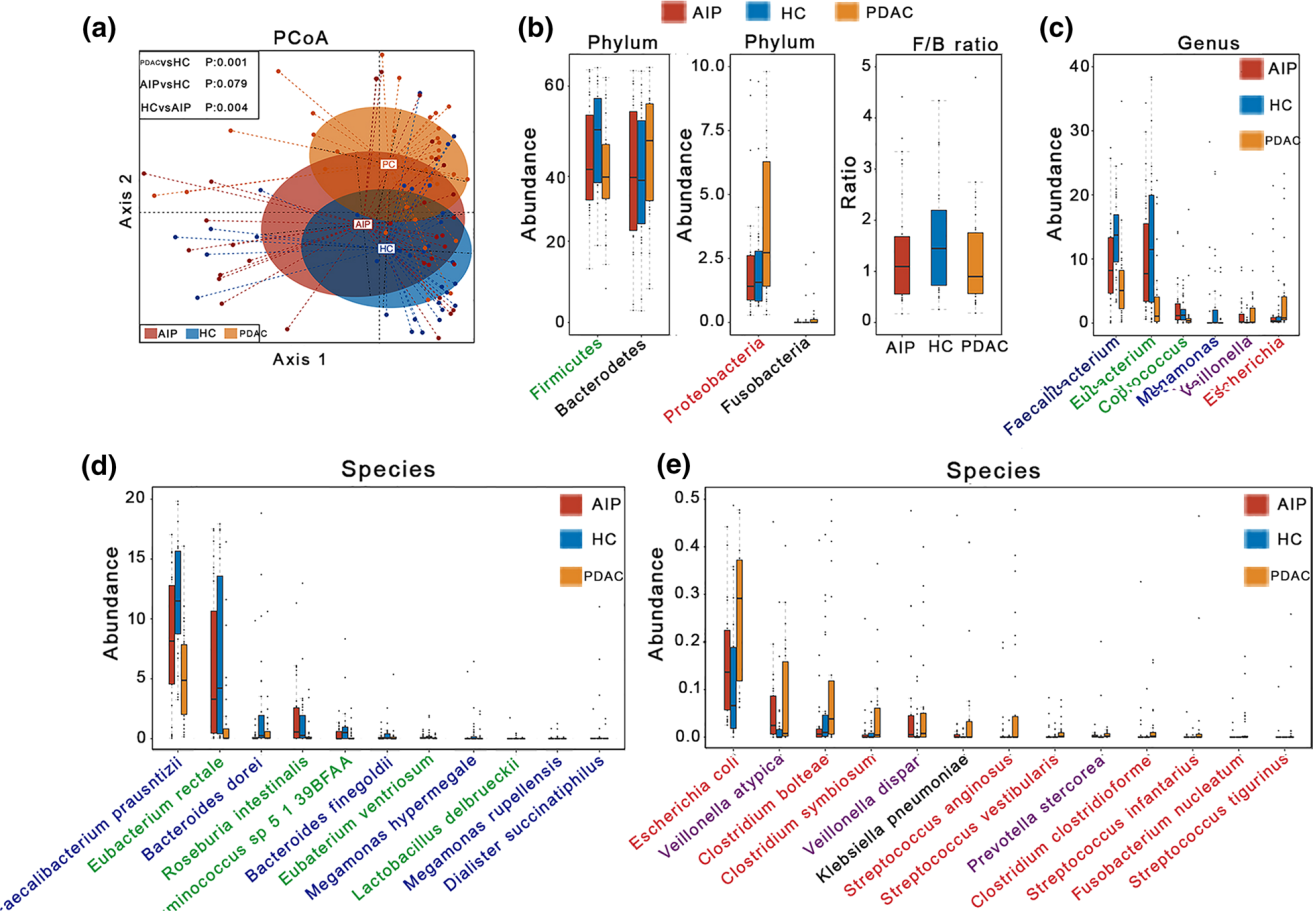
Alterations in the gut microbiota among patients with PDAC and AIP. **a** Principal coordinate analysis (PCoA) based on Bray–Curtis distance at the species level. Each data point represents an individual sample. P-value was calculated by PERMANOVA. **b** Comparison of the gut microbiota among PDAC, AIP and HC groups at the phylum level and box and whisker plot of Firmicutes to Bacteroidetes ratios. **c–e** Species enriched in healthy controls and species enriched in case groups, respectively. The phylum, genus and species levels are colored as follows: purple, both enriched in PDAC and AIP; blue, both depleted in PDAC and AIP; red, enriched in PDAC only; green, depleted in PDAC only. Significance was set at adjusted p-value < 0.2 (FDR-corrected Kruskal–Wallis test) and p-value (Steel–Dwass test) < 0.05. Only taxa with adjusted p-value < 0.2 (except Bacteroidetes) are shown. Steel–Dwass test for pairwise comparisons following FDR-corrected Kruskal–Wallis test. PDAC: pancreatic ductal adenocarcinoma; AIP: autoimmune pancreatitis; HC: healthy controls

To investigate the microbial compositional differences among groups, we performed principal coordinate analysis (PCoA) (Fig. 1a). The results revealed that axis 2 discriminated most of the HC samples from most of the PDAC samples (PERMANOVA, p = 0.001). Moreover, PERMANOVA analysis revealed that the overall microbial compositions differed significantly between PDAC and AIP samples (PERMANOVA, p = 0.004). However, samples in the AIP and HC groups tended to cluster together with a p-value > 0.05 (PERAMANOVA) (Fig. 1a). Here, to evaluate how diabetes and hypertension affect the microbial compositional differences between participants, a permutation test with `*adonis2`* function (R package: vegan) was performed and results showed no statistically significant effect of both metabolic disorders on the microbial compositional differences (diabetes: R^2^ = 0.009, p = 0.648; hypertension: R^2^ = 0.009, p = 0.334).In addition, no statistically significant differences in α-diversity were observed between any two of the three groups as indicated by the Shannon index, Simpson index, richness and evenness (Additional file 7: Figure S1).

We further investigated and compared the relative microbial community abundances at every level (Fig. 1b–e, Additional file 3). At the phylum level (Fig. 1b and Additional file 3), Firmicutes and Bacteroidetes remained the dominant phyla, followed by Proteobacteria in the case groups; However, we observed a significantly decreased prevalence of Firmicutes (p = 0.0013, Steel–Dwass test) and increased prevalence of Proteobacteria (p = 0.013, Steel–Dwass test), especially Gammaproteobacteria (p = 0.0062, Steel–Dwass test), among PDAC samples, but not among AIP samples. Notably, colorectal cancer-associated Phylum Fusobacteria [22, 23] were also found differentially enriched between HC and PDAC groups with an adjusted p-value of < 0.2 (Benjamini–Hochberg corrected Kruskal–Wallis test), showing a relative increase among PDAC samples (p = 0.075, Steel–Dwass test). At the genus level (Fig. 1c and Additional file 3), *Megamonas*, *Faecalibacterium, Eubacterium* and *Coprococcus* were significantly decreased in the PDAC group, while only *Megamonas* and *Faecalibacterium* were significantly decreased in the AIP group. The genus *Veillonella* was significantly increased in both the AIP and PDAC groups, while *Escherichia* was significantly increased only in the PDAC group. We identified 24, 12 and 15species differentially enriched between the PDAC/HC, AIP/HC and PDAC/AIP groups, respectively (Additional file 3). We observed a depletion of SCFA-producing bacteria, including *Faecalibacterium prausnitzii, Eubacterium rectale*, *Roseburia intestinalis*, and *Ruminococcussp 5_1_39BFAA*, along with an enrichment of *Escherichia coli*, *Fusobacterium nucleatum* and some *Clostridium spp* among PDAC individuals (Fig. 1d and e). However, only *Faecalibacterium prausnitzii* was decreased among AIP individuals. *Megamonas spp*, *Veillonella atypica, Veillonella parvula* and *Prevotella stercorea* were enriched in both PDAC and AIP samples (Fig. 1d and e). Although we also annotated several species of viruses and archaea, no statistically significant differences were observed among the three groups (data not shown).

### Metagenomic species profiling for PDAC and AIP groups

**Fig. 2:**
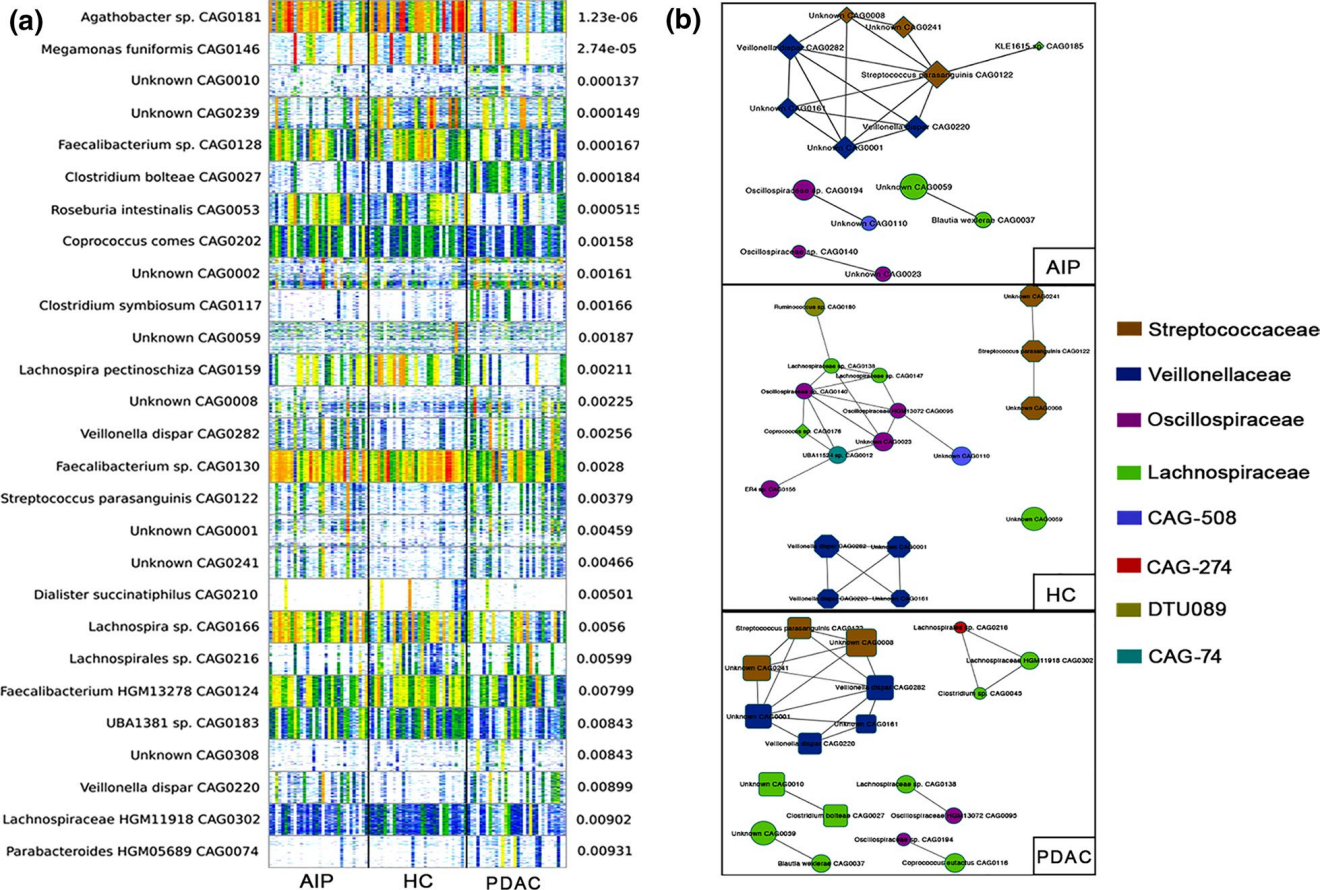
Differentially abundant CAGs in the three groups. **a** Heatmap of CAGs with a p-value < 0.05determined by the Kruskal–Wallis testis shown in rows. Gene abundance is indicated by color gradient (white represents “undetected”, red represents “most abundant”). The p-value is shown on the right. **b** Abundance-based CAG co-abundance correlation network enriched in PC group (left), AIP group (media) and AIP group (right). The node size was proportional to the enrichment extent. The node shape was as follows: rectangle, PDAC enriched; rhombus, AIP enriched; diamond, case enriched; circle, HC enriched. Nodes are colored by family level. PDAC: pancreatic ductal adenocarcinoma; AIP: autoimmune pancreatitis; HC: healthy control

To better learn about the microbial compositional characterization of PDAC and AIP individuals, we performed metagenomic species (MGS) profiling to complement MetaPhlAn2.0. Results showed among all the MGSs (also called co-abundance gene groups(CAGs)) with genes > 500, a total of 21 CAGs were differently enriched between groups with an adjusted p-value < 0.2 (Benjamini–Hochberg corrected Kruskal–Wallis test); And 18, 9 and 10 of the 21 CAGs differed significantly between the PDAC/HC, AIP/HC and PDAC/AIP groups, respectively (Fig. 2a and Additional file 4), In detail, *Faecalibacterium sp*. and *Roseburia intestinalis* were decreased, and *Clostridium bolteae* and *Clostridium symbiosum* were increased in only the PDAC group. *Megamonas funiformis* and *Dialister succinatiphilus* were decreased, and *Veillonella dispar* and *Streptococcus parasanguinis* were increased in both the PDAC and AIP groups. It’s interesting to note that *Veillonella dispar* and *Streptococcus parasanguinis* were mostly oral species [24]. In addition, 7 species were unknown because less than 50% of the genes were assigned a specific species, indicating that unknown microbial organisms may be associated with the PDAC and AIP statuses.

To gain new insights into the possible interactions between the differentially abundant microbes, a co-occurrence analysis [16] was performed (Fig. 2b). In healthy controls, species from Lachnospiraceae and Oscillospiraceae tended to cluster and have more correlations, while in the case samples, species from Streptococcaceae and Veillonellaceae tended to cluster. Additionally, more unknown CAGs played roles in the network in the case samples. When comparing the PDAC and AIP samples, the correlations in the PDAC group were closer and more complex; CAG0282 played a central role in the network of the PDAC samples and CAG0122 in the AIP group. Although these results showed a profound dysbiosis in both the gut microbial compositions and interactions among cases, large cohort studies are needed to further elucidate these interactions.

### Overall analysis of microbial community functions in the PDAC and AIP groups

**Fig. 3:**
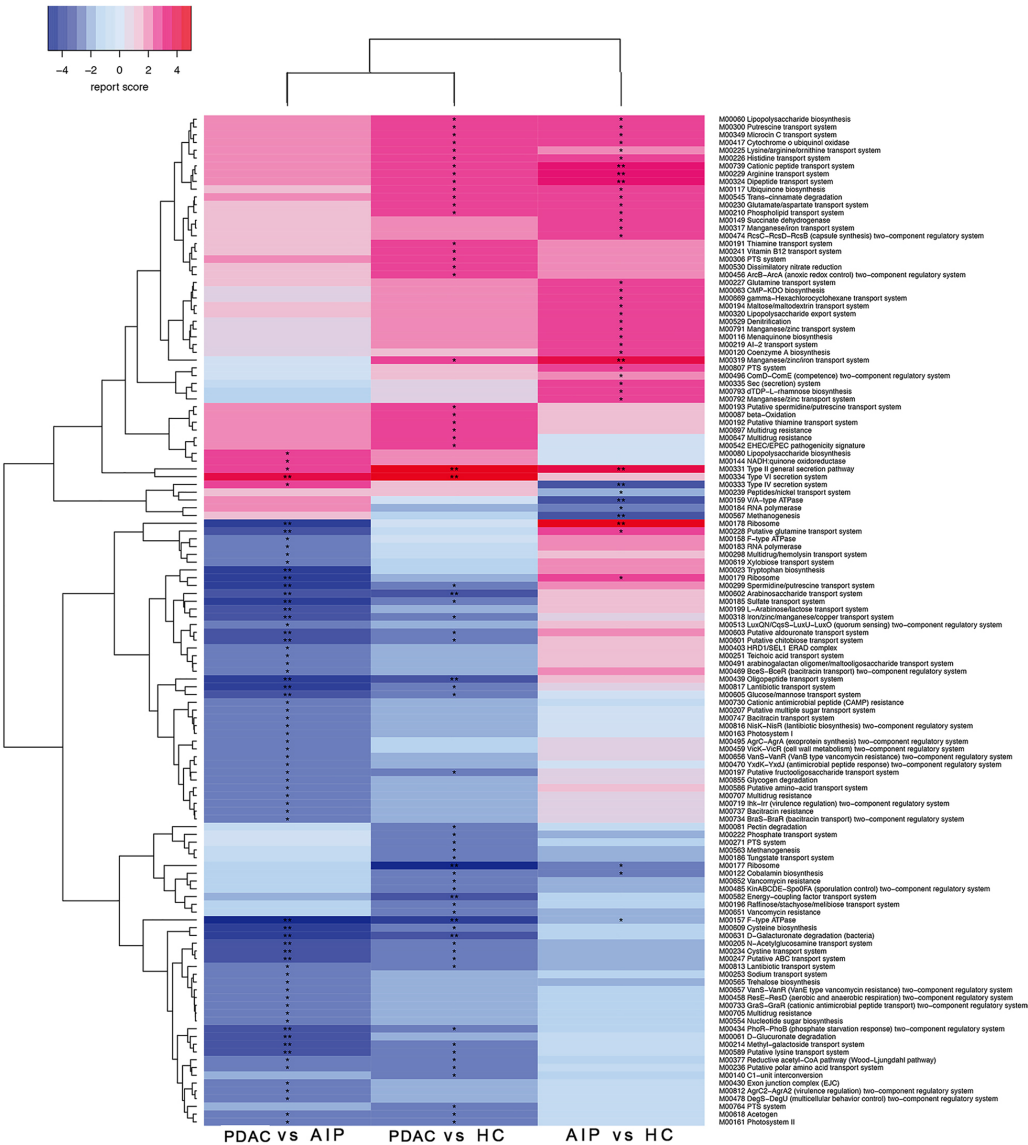
Differentially enriched KO modules in the PDAC, AIP and HC groups. Modules with a reporter score > 2.3 (the former enriched) or < -2.3 (the latter were enriched) are shown. *reporter score > 2.3 or < − 2.3; **, reporter score > 3 or < 3. PDAC: pancreatic ductal adenocarcinoma; AIP: autoimmune pancreatitis; HC: healthy control

To improve our knowledge of gut microbial functions among PDAC and AIP individuals, we identified the differentially enriched KO modules among groups according to reporter score (Fig. 3 and Additional file 5). Results showed the gut microbiota of PDAC patients displayed a higher potential to degrade fatty acids and a notably lower metabolic capacity to synthesize short-chain fatty acids (SCFAs), especially acetate and butyrate. Among PDAC individuals, the Wood-Ljungdahl pathway (the classic acetate-producing pathway) was depleted; in addition, pyruvate is an important intermediate product of SCFA biosynthesis [18, 25], whereas the potential to produce pyruvate through D-galacturonate and glucuronate degradation was decreased; furthermore, the PDAC samples exhibited lower potential for transporting many saccharides, such as fructooligosaccharide and glucose.

Another notable result among PDAC samples was that we observed a possible higher potential for putrescine and spermidine transportation. Then we performed further analysis per HUMAnN2 and results showed two (superpathway of arginine and polyamine biosynthesis, superpathway of polyamine biosynthesis I) of the three identified MetaCyc pathways involved in polyamine biosynthesis increased in the guts of PDAC patients and *E. coli* may be the main contributary species (Additional file 7: Figure S2). However, polyamines were not detected in case feces in our lab (data not shown), which may be due to the low sensitivity of our method or a considerable degradation of polyamines.

Other observations among PDAC samples included increased type II general secretion pathway (T2S), type VI secretion system (T6S), lipopolysaccharide (LPS) biosynthesis and upregulated M00210which can increase gram-negative bacterial vitality through contributing to asymmetric lipid distribution [26]. In contrast, among AIP samples, we only found significantly increased T2S, M00335, M00210 and decreased T4S, but no significant changes were observed in SCFA and polyamine production compared to HC samples.

### Downregulated butyrate production in feces of patients with PDAC, but not in feces of patients with AIP as revealed by bioinformatic analysis and gas chromatography

**Fig. 4:**
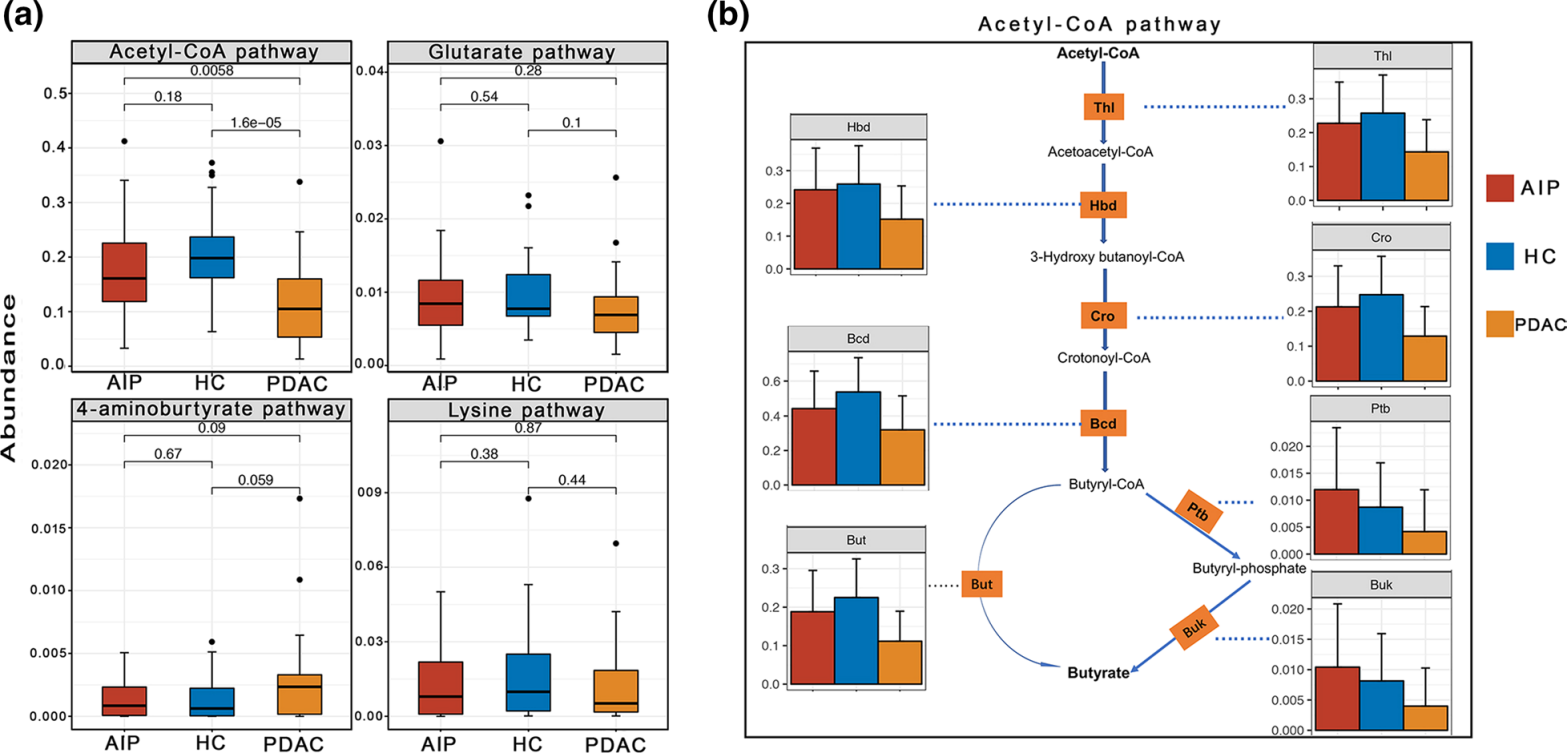
Box-and-Whisker plot of four pathways for butyrate synthesis (**a**) and bar plot of involved genes in acetyl-CoA pathway (**b**). p-value shown in (**a**) was determined by Steel–Dwass test for pairwise comparison following FDR-corrected Kruskal–Wallis test. Pathways are shown as described by Vital et al. [14]

**Fig. 5:**
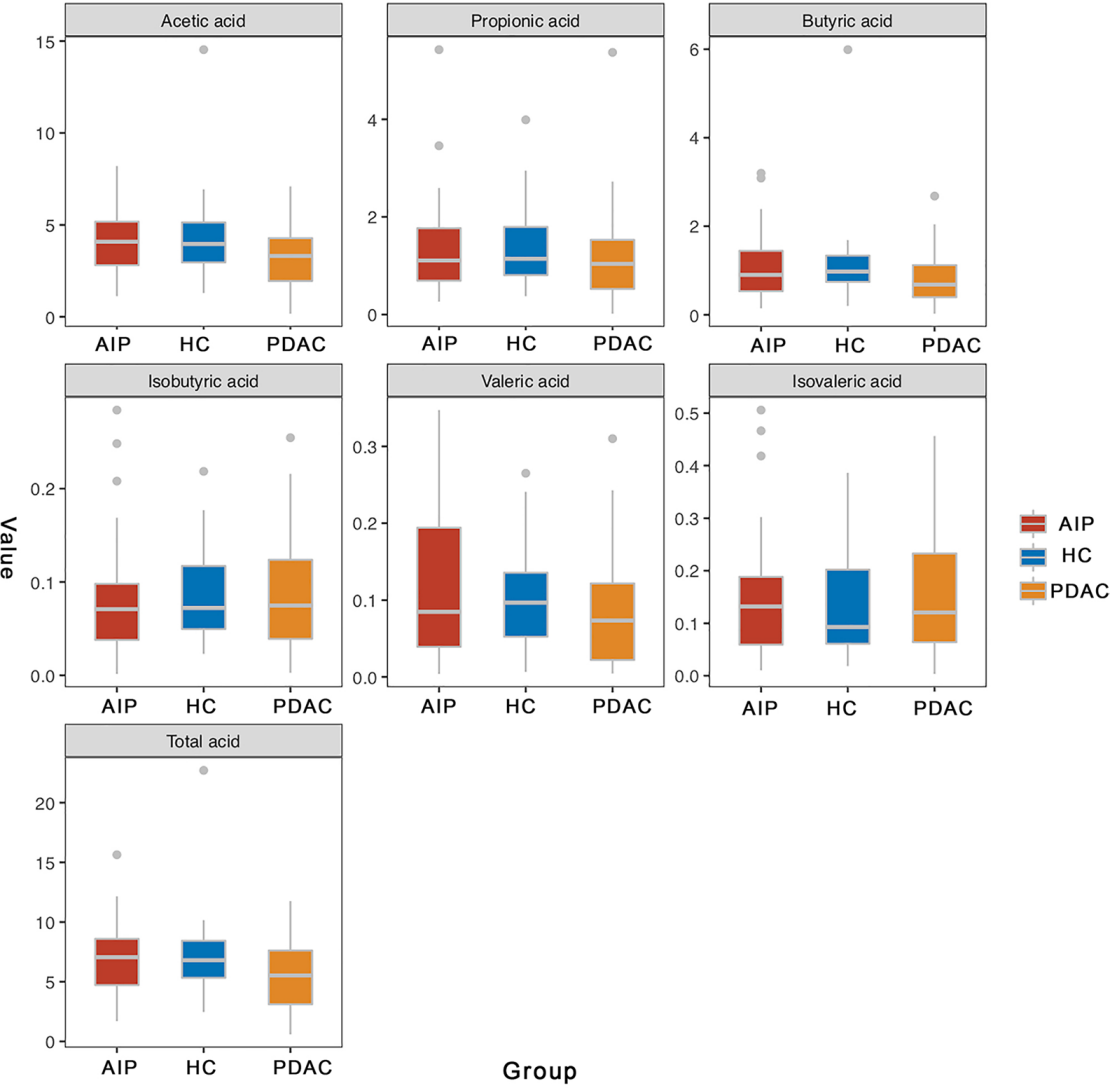
Butyrate was downregulated only in feces of PDAC patients as revealed by gas chromatography analysis. Shown are relative content of acetic acid, propionic acid, butyric acid, isobutyric acid, valeric acid, isovaleric acid and total acid in PDAC, AIP and HC groups. PDAC: pancreatic ductal adenocarcinoma; AIP: autoimmune pancreatitis; HC: healthy controls CI: confidence interval

Considering a profound change was observed in butyrate-producing bacteria among PDAC samples, we further investigated the differences in the butyrate-producing pathways and involved genes described by Vital et al. [18] (Fig. 4a and b, Additional file 7: Figure S3 and Additional file 6). Results showed among PDAC samples three of the four main pathways displayed a downward trend, especially the acetyl-CoA pathway (PDAC vs HC, p = 1.6e-05); In addition, all genes involved in this pathway were downregulated, especially gene *HBD, BUK, PTB* with a p value < 0.05 compared with HC samples. Here, the 4-aminobutyrate pathway was slightly increased, although not statistically, and the involved gene, *X4HBT*, was significantly increased in the PDAC group which may be associated with the increase in *Fusobacterium* [18]. Then we performed quantitative detection of fecal butyrate among PDAC samples and results further confirmed a significate reduction of butyrate content compared with HC samples (p = 0.02, Fig. 5), along with a downtrend in acetate content (p = 0.12, Fig. 5). In contrast, although butyrate-producing *Faecalibacterium prausnitzii* [18] was decreased among AIP patients, AIP and HC patients did not differ significantly in any of the four pathways or their involved genes or butyrate content in feces, which was consistent with the idea that butyrate producers were a functional cohort, and the gut can partially maintain butyrate production [18].

### Bacterial markers that identify patients with PDAC

**Fig. 6:**
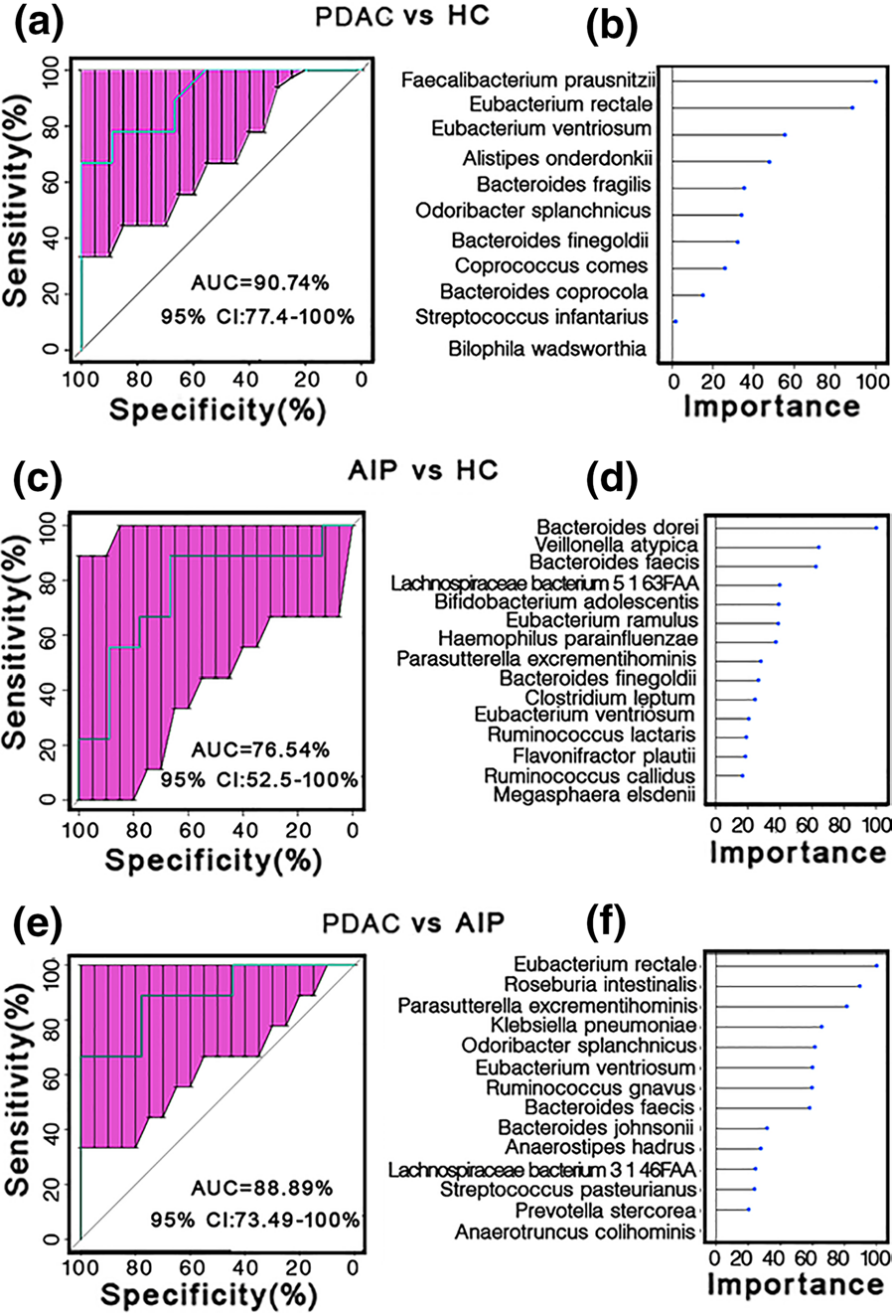
Microbial biomarkers classify PDACC patients from healthy controls and AIP patients. **a**, **c**, **e** ROC curves for testing cohort are shown. **b**, **d**, **f** Shown are the importance of the selected biomarkers. PDAC vs AIP (**a** and **b**), AIP vs HC (**c** and **d**), PDAC vs AIP (**e** and **f**). PDAC: pancreatic ductal adenocarcinoma; AIP: autoimmune pancreatitis; HC: healthy controls CI: confidence interval

To test whether the microbiota composition can distinguish PDAC patients from either healthy controls or AIP patients, a classifier was constructed using the Random Forest method. Here, 11, 15 and 14 species were selected as biomarkers to discriminate PDAC/HC, AIP/HC and PDAC/AIP individuals, respectively (Fig. 6b, d and f). Interestingly, *Eubacterium rectale*, *Eubacterium ventrisum* and *Odoribacter splanchnicus* was among the most important biomarkers in distinguishing PDAC from HC and from AIP individuals. To increase the robustness of the identified biomarkers, we performed LEfSe analysis and results showed that most of the identified biomarkers exhibit biological significance with a linear discriminant analysis (LDA) score > 2 (Additional file 7: Figure S4). ROC analysis showed an AUC of 90.74% (95% confidence interval [CI] 77.4–100%), 88.89% (95% CI 73.49–100%) and 76.54% (95% CI 52.5–100%) in the PDAC/HC, PDAC/AIP and AIP/HC cohort, respectively (Fig. 6a, c, e), indicating a potential role of the gut microbial biomarkers as a non-invasive screening strategy for PDAC diagnosis and PDAC/AIP discrimination but not for AIP/HC discrimination.

## Discussion

The gut microbiota has been associated with many human diseases including gastrointestinal cancer [27, 28]. In this study, we revealed that patients with PDAC harbored an evidently different microbiota in their guts compared with healthy controls, whereas AIP patients displayed relatively mild alterations in both composition and function. Furthermore, we provided a possible new method of non-invasively identifying PDAC from HC or AIP, although large cohort studies are needed for further validation.

In our study, the samples of PDAC displayed significantly reduced phylum Firmicutes, mainly due toa significant reduction in butyrate-producing bacteria, including *Faecalibacterium prausnitzii*, *Eubacterium rectale* and *Roseburia intestinali*, which are important in gut health maintenance [18]. *Faecalibacterium prausnitzii,* a major commensal gut bacterium, has an important anti-inflammatory property by blocking NF-κB activation and interleukin-8 (IL-8) and is often underrepresented in many inflammatory diseases [29]. Pieter et.al found *Eubacterium rectale* and *Roseburia intestinalis* can play protective roles by colonizing mucins [30] through which they occupy an ecological niche and, together with antimicrobials, limit pathogen translocation. Moreover, *Roseburia intestinalis* has been recently suggested to be positively associated with tight-junction integrity in the gut [31]. These results together suggest an inflammatory status and dysregulated gut barrier in the PDAC gut, which provides a potential means for opportunistic pathogen translocation, thus affecting the pancreatic oncogenesis and tumor progression.

It’s worth noting that Gammaproteobacteria (especially *E. coli*), which consists of a large number of pathogenic bacteria, was found a significant increase among PDAC samples. Some species of Proteobacteria, including *E. coli*, can increase the mutation in the infected cells, at least in part through releasing colibactin and cytolethal distending toxin (CDT) [32]. Colibactin and CDT are also associated with *E. coli*’s survival in the microenvironment by killing competitors [32]. Furthermore, functional analysis suggested that *E. coli* in the PDAC participants exhibits a phenotype of enhanced virulence. To be specific, T2S, which has been reported only in Proteobacteria, especially at a higher incidence [33], was predicted an increase among PDAC samples, which can enhance virulence by allowing heat-labile toxin transportation into extracellular and even host cells [33]; in addition, increased T6S among patients with PDAC has been reported in *E. coli*, which is associated with bacterial virulence and is likely to play an important role in favoring *E. coli* growth [34]. Oral-resident *Fusobacterium nucleatum* [35], which has a suggested role in colorectal cancer [22], was also found enriched among PDAC samples in our study. Notably, Fusobacterium colonization was indeed identified in pancreatic cancer tissue and in adjacent normal tissue in a previous study [36]. In addition, existing evidence in literature supported that by interacting with endothelial cell and epithelial cells through two main virulence factors (FadA and Fap2), *Fusobacterium nucleatum* can promotes a proinflammatory and immunosuppressive tumor microenvironment, thereby promoting tumor growth and progression (eg. colorectal cancer) [37]. Intriguingly, prevalence of Fusobacterium in pancreatic cancer tissue was described to be associated with a worse prognosis by Mitsuhashi K et al. [36]. Altogether, *Fusobacterium nucleatum* is a potential pathogenic bacterium for pancreatic cancer and the underlying molecular mechanism is worthy of further validation.

Consistent with the reduction of butyrate-producing bacteria and downregulation of butyrate pathways, we detected a significant decrease of butyrate content in fecal samples of PDAC. The butyrate is the main preferred energy source of colonic epithelial cells (ECs), as the essential part of first line of defense, the normal energy supply of colonic ECs is the basis for its’ normal barrier function [18, 38]. In addition, butyrate is an important anti-inflammatory product of the gut microbiota [18, 38], leading to its key role in maintaining gut homeostasis. Importantly, butyrate is also a histone deacetylase inhibitor (HDACi) [34]. Studies have shown that abnormal histone deacetylation is associated with malignant tumors and HDACi can inhibit cancer progression through remodeling histone acetylation [39]. As an HDACi, in vitro studies have indicated that butyrate can inhibit the growth of colon, prostate, and cervical carcinomas by inducing apoptosis, differentiation and cell-cycle arrest [39]. Interestingly, butyrate is a preferred energy substrate for normal colonocytes instead of inhibiting cell growth, whereas butyrate concentrations are much higher in cancer cells by acting as an HDACi [25]. What’s more, in vitro studies have indicated that butyrate may play a role in inhibiting pancreatic cancer invasion by downregulating β4 integrin expression [40]. Above all, our study provides new support for the link between butyrate and PDAC, but how butyrate is involved in the progression of PDAC needs more studies.

We also found a possible higher potential to produce polyamines mainly due to the increased prevalence of *E. coli* in the guts of patients with PDAC. Dysregulated polyamine levels have been associated with toxic effects and carcinogenesis, and increased polyamine levels in urine and blood specimens have been found among other cancers, such as skin cancer [41]. However, we failed to measure polyamines in our subjects, whether there is a relationship between polyamines and PDAC needs further study.

Here, we identified a combination of fecal microbial biomarkers that could distinguish patients with PDAC from healthy controls (AUC = 90.74%) with relatively high specificity. However, we failed to perform validation study in the cohort of early PDAC patients due to the limited sample size. In addition, we also identified a microbial combination that could distinguish PDAC from AIP individuals (AUC = 88.89%), Considering the difficulty in distinguishing PDAC from AIP, the result is promising. However, large studies are needed to further investigate whether the established classifier here will be clinically helpful. As we depicted, fecal microbial species failed to discriminate AIP patients from healthy controls (AUC = 76.54%), which is consistent with the mild gut microbial disturbance in AIP samples. Differences in gut microbial alterations between the PDAC and AIP groups may explain the low cancer rate in AIP patients.

## Conclusions

Our study revealed an obviously disturbed fecal microbial composition and function among PDAC individuals and notably, butyrate-producing bacteria and butyrate concentration were significantly downregulated, suggesting an association between the gut microbiota and PDAC. Fecal bacterial species and butyrate may be helpful biomarkers in PDAC diagnosis and differentiating PDAC from AIP patients. All in all, these results indicate that the specific mechanisms and roles of the gut microbiota in PDAC patients are worth to be further investigated.

## Supplementary Information

**Additional file 1: Table S1.** Statistics for the Illumina metagenomic sequencing data.

**Additional file 2: Table S2**. Denovo assembly of high-quality metagenomic shotgun sequencing data.

**Additional file 3: Table S3.** Differentially enriched gut microbiota at every level between groups using MetaPhlAn2 method. Taxon with a p. adj < 0.2 (FDR-corrected Kruskal–Wallis test) were shown. FDR-corrected Kruskal–Wallis test was followed by Steel–Dwass test for pairwise comparisons. Threshold of p-value (Steel–Dwass test) was set at 0.05.

**Additional file 4: Table S4.** Differentially enriched CAGs between PDAC/HC, AIP/HC or PDAC/AIP groups.

**Additional file 5: Table S5.** Differentially enriched KO modules in PDAC, AIP and HC groups. modules with a repoter score > 2.3 (the former enriched) or < − 2.3 (the latter were enriched) were shown.

**Additional file 6: Table S6.** Comparison of genes involved in butyrate synthesis in acetyl-CoA pathway, aminobutyrate pathway, glutarate pathway and lysine pathway. FDR-corrected Kruskal–Wallis test followed by Steel–Dwass test for pairwise comparisons.

**Additional file 7: Figure S1.** Box-and-whisker plot of alpha diversity indices, including diversity (Shannon, Simpson), community richness and evenness. P-value was determined by the Kruskal–Wallis test followed by Steel–Dwass test for multiple comparisons. PDAC: pancreatic ductal adenocarcinoma; AIP: autoimmune pancreatitis; HC: healthy controls. Figure S2. Analysis of three identified MetaCyc pathways involved in polyamine biosynthesis using HUMAnN2. Figure S3 Bar plot of genes involved in butyrate synthesis in the aminobutyrate, glutarate and lysine pathways. Figure S4 Differentially abundant bacterial species with a statistical and biological significance as revealed by LEfSe analysis between PDAC/HC, AIP/HC or PDAC/AIP groups. PDAC: pancreatic ductal adenocarcinoma; AIP: autoimmune pancreatitis; HC: healthy controls; LDA: linear discriminant analysis; LEfSe: LDA effect size.

## Data Availability

The datasets used and/or analysed during the current study are available from the corresponding author on reasonable request.

## Abbreviations

PDAC: Pancreatic ductal adenocarcinomas
AIP: Autoimmune pancreatitis
IgG4: Immunoglobulin G4
HCs: Healthy controls
RPKM: Per kilobase per million
MGS: Metagenomic species
CAGs: Co-abundance gene groups
KEGG: Kyoto Encyclopedia of Genes and Genomes
KO: KEGG ortholog
SCFA: Short-chain fatty acids
ROC: Receiver operation characteristic
AUC: Area under the ROC curve
PCoA: Principal coordinate analysis
PERMANOVA: Permutational multivariate analysis of variance
ECs: Epithelial cells
HDACi: Histone deacetylase inhibitor
