# Supplementary material for: The fecal microbiota of patients with pancreatic ductal adenocarcinoma and autoimmune pancreatitis characterized by metagenomic sequencing
Source: J Transl Med. 2021 May 18;19:215. doi: 10.1186/s12967-021-02882-7 (PMC8130326; doi:10.1186/s12967-021-02882-7)
Supplement: Supplementary file 7 — Additional file 7: Figure S1. Box-and-whisker plot of alpha diversity indices, including diversity (Shannon, Simpson), community richness and evenness. P-value was determined by the Kruskal–Wallis test followed by Steel–Dwass test for multiple comparisons. PDAC: pancreatic ductal adenocarcinoma; AIP: autoimmune pancreatitis; HC: healthy controls. Figure S2. Analysis of three identified MetaCyc pathways involved in polyamine biosynthesis using HUMAnN2. Figure S3 Bar plot of genes involved in butyrate synthesis in the aminobutyrate, glutarate and lysine pathways. Figure S4 Differentially abundant bacterial species with a statistical and biological significance as revealed by LEfSe analysis between PDAC/HC, AIP/HC or PDAC/AIP groups. PDAC: pancreatic ductal adenocarcinoma; AIP: autoimmune pancreatitis; HC: healthy controls; LDA: linear discriminant analysis; LEfSe: LDA effect size. [file 12967_2021_2882_MOESM7_ESM.docx]

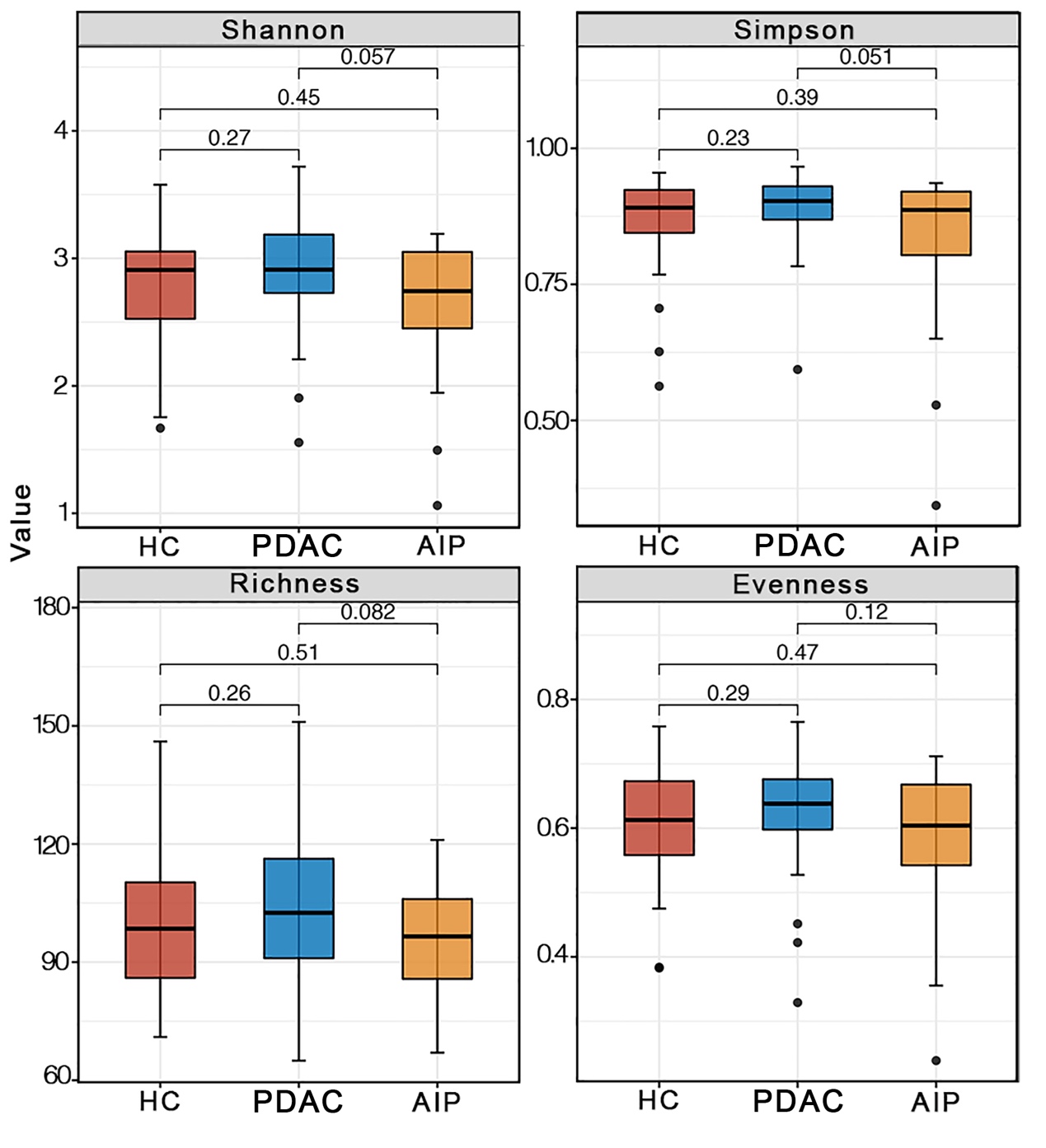


**Figure S1 Box-and-whisker plot of alpha diversity indices, including diversity (Shannon, Simpson), community richness and evenness**

P-value was determined by the Kruskal-Wallis test followed by Steel-Dwass test for multiple comparisons. PDAC: pancreatic ductal adenocarcinoma; AIP: autoimmune pancreatitis; HC: healthy controls.


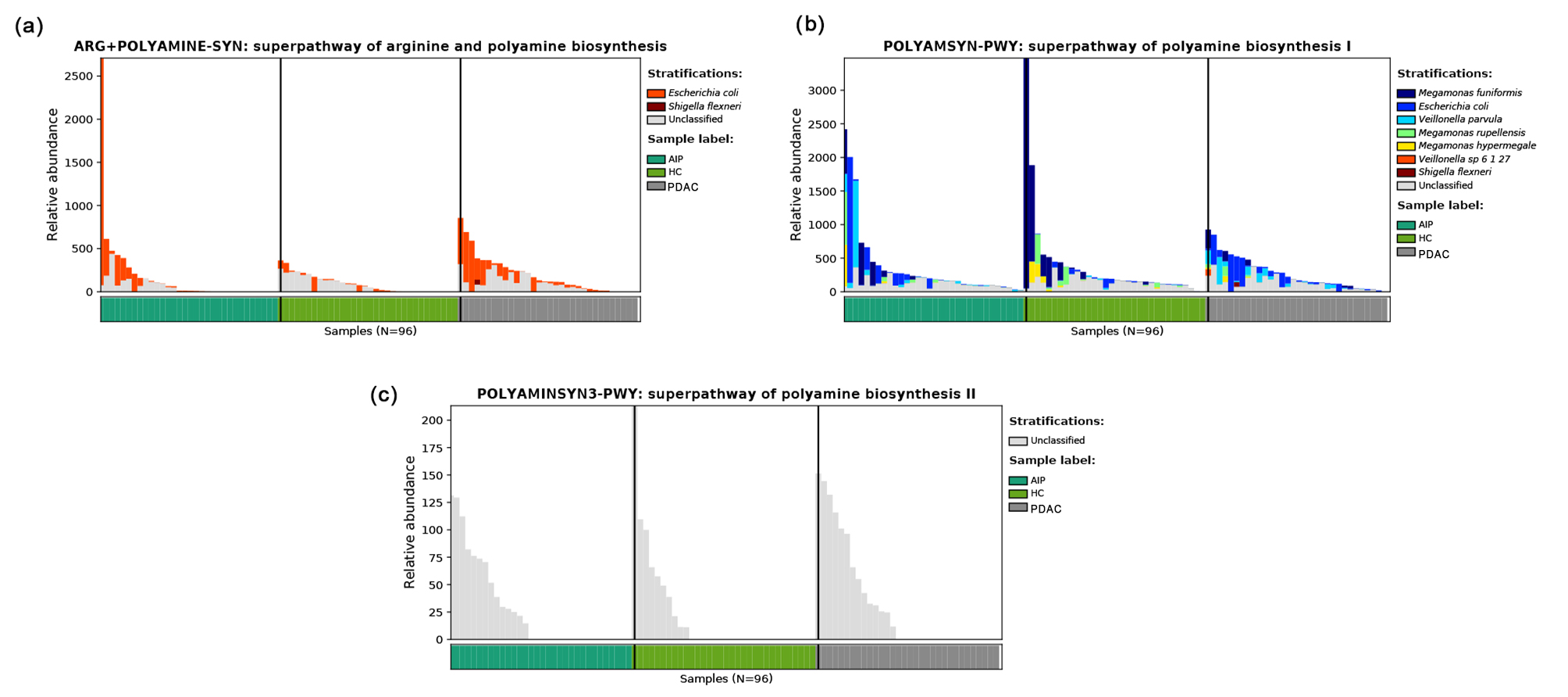


**Figure S2 Analysis of three identified MetaCyc pathways involved in polyamine biosynthesis using HUMAnN2**

**
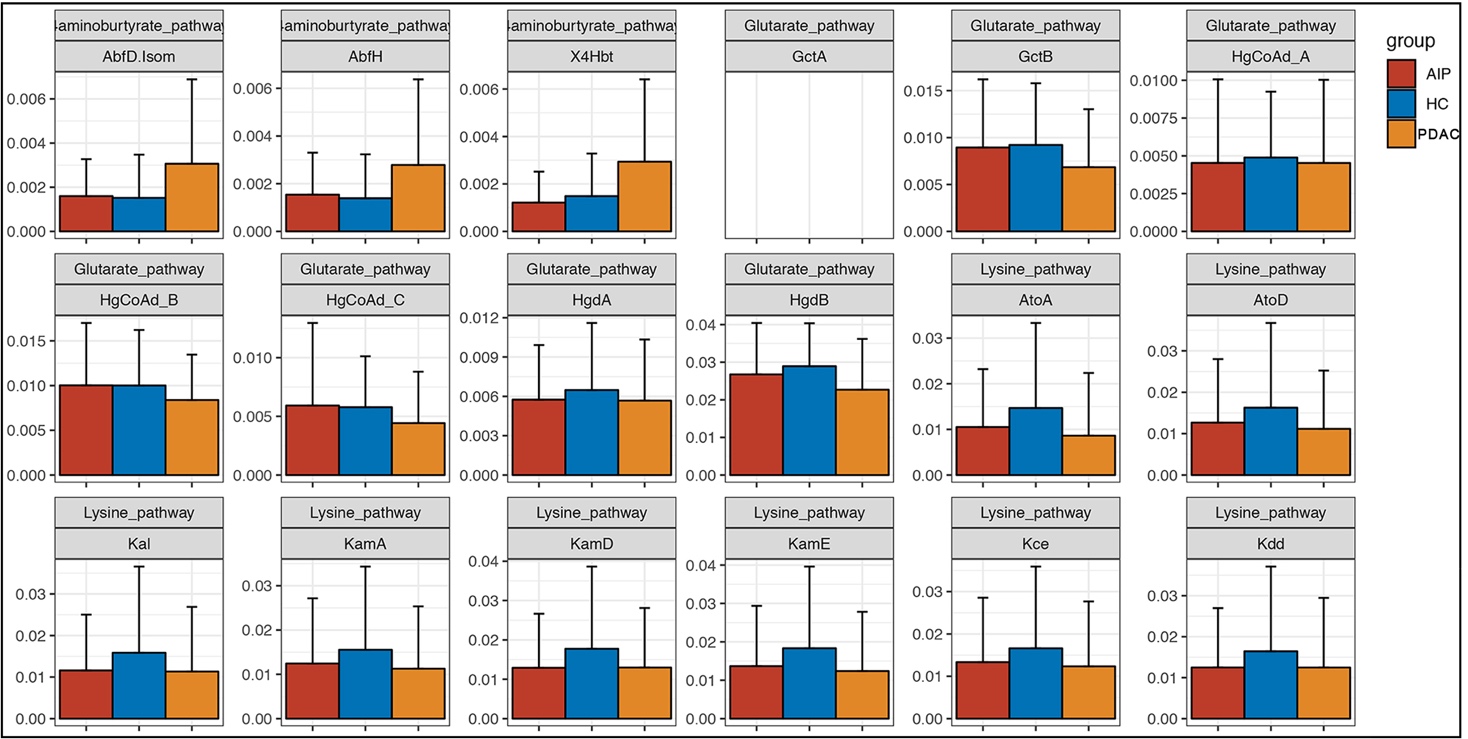
**

**Figure S3 Bar plot of genes involved in butyrate synthesis in the aminobutyrate, glutarate and lysine pathways**

**
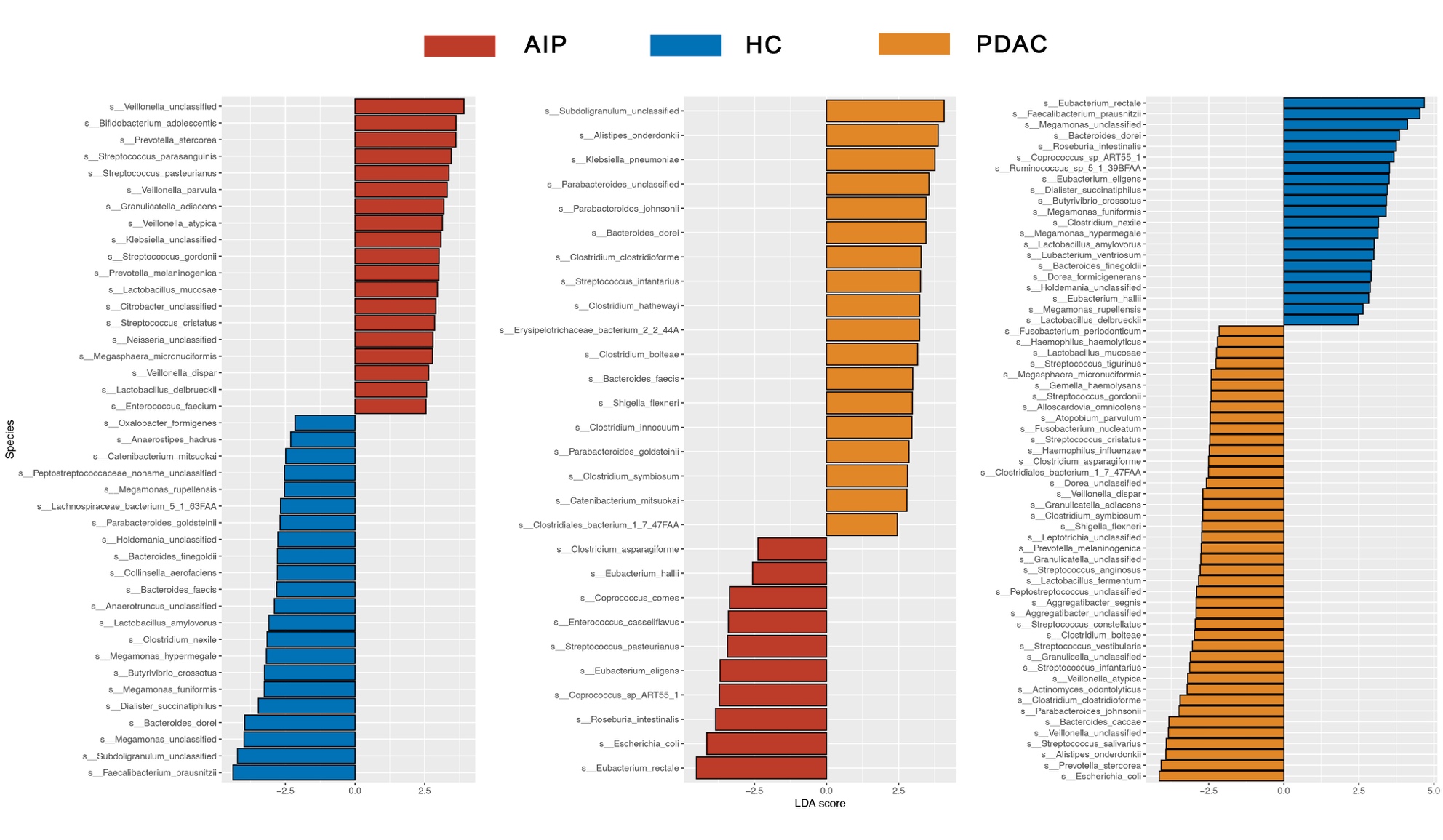
**

**Figure S4 Differentially abundant bacterial species with a statistical and biological significance as revealed by LEfSe analysis between PDAC/HC, AIP/HC or PDAC/AIP groups. PDAC: pancreatic ductal adenocarcinoma; AIP: autoimmune pancreatitis; HC: healthy controls; LDA: linear discriminant analysis; LEfSe: LDA effect size.**
